# Cost-effectiveness of Installing Barriers at Bridge and Cliff Sites for Suicide Prevention in Australia

**DOI:** 10.1001/jamanetworkopen.2022.6019

**Published:** 2022-04-05

**Authors:** Piumee Bandara, Jane Pirkis, Angela Clapperton, Sangsoo Shin, Lay San Too, Lennart Reifels, Sandersan Onie, Andrew Page, Karl Andriessen, Karolina Krysinska, Anna Flego, Marisa Schlichthorst, Matthew J. Spittal, Cathrine Mihalopoulos, Long Khanh-Dao Le

**Affiliations:** 1 Translational Health Research Institute, Western Sydney University, Sydney, New South Wales, Australia; 2 Centre for Mental Health, Melbourne School of Population and Global Health, The University of Melbourne, Melbourne, Victoria, Australia; 3 Black Dog Institute, University of New South Wales, Sydney, New South Wales, Australia; 4 Emotional Health for All Foundation, Jakarta, Indonesia; 5 Deakin Health Economics, Institute for Health Transformation, School of Health and Social Development, Deakin University, Burwood, Victoria, Australia

## Abstract

**Importance:**

Installation of barriers has been shown to reduce suicides. To our knowledge, no studies have evaluated the cost-effectiveness of installing barriers at multiple bridge and cliff sites where suicides are known to occur.

**Objective:**

To examine the cost-effectiveness of installing barriers at bridge and cliff sites throughout Australia.

**Design, Setting, and Participants:**

This economic evaluation used an economic model to examine the costs, costs saved, and reductions in suicides if barriers were installed across identified bridge and cliff sites over 5 and 10 years. Specific and accessible bridge and cliff sites across Australia that reported 2 or more suicides over a 5-year period were identified for analysis. A partial societal perspective (including intervention costs and monetary value associated with preventing suicide deaths) was adopted in the development of the model.

**Interventions:**

Barriers installed at bridge and cliff sites.

**Main Outcomes and Measures:**

Primary outcome was return on investment (ROI) comparing cost savings with intervention costs. Secondary outcomes included incremental cost-effectiveness ratio (ICER), comprising the difference in costs between installation of barriers and no installation of barriers divided by the difference in reduction of suicide cases. Uncertainty and sensitivity analyses were undertaken to examine the association of changes in suicide rates with barrier installation, adjustments to the value of statistical life, and changes in maintenance costs of barriers.

**Results:**

A total of 7 bridges and 19 cliff sites were included in the model. If barriers were installed at bridge sites, an estimated US $145 million (95% uncertainty interval [UI], $90 to $160 million) could be saved in prevented suicides over 5 years, and US $270 million (95% UI, $176 to $298 million) over 10 years. The estimated ROI ratio for building barriers over 10 years at bridges was 2.4 (95% UI, 1.5 to 2.7); the results for cliff sites were not significant (ROI, 2.0; 95% UI, −1.1 to 3.8). The ICER indicated monetary savings due to averted suicides over the intervention cost for bridges, although evidence for similar savings was not significant for cliffs. Results were robust in all sensitivity analyses except when the value of statistical life-year over 5 or 10 years only was used.

**Conclusions and Relevance:**

In an economic analysis, barriers were a cost-effective suicide prevention intervention at bridge sites. Further research is required for cliff sites.

## Key Points

**Question:**

Are barriers installed for suicide prevention at bridge and cliff sites cost-effective?

**Findings:**

This economic evaluation found that barriers installed at multiple bridge sites across Australia were a cost-saving intervention with a return of US $2.40 for every US $1 invested over 10 years. Evidence was not significant for cliff sites, highlighting the need for further research.

**Meaning:**

These results suggest that barriers are a cost-effective measure associated with reduced rates of suicide at bridge sites; their installation is a warranted strategy for suicide prevention.

## Teaser

This economic evaluation examines the cost-effectiveness of barrier installation as a suicide prevention measure at bridges and cliff sites in Australia.

## Introduction

Suicide is a significant public health issue worldwide, resulting in premature mortality with long-term effects on families, friends, and society at large. In Australia, more than 3000 people died by suicide in 2020, and suicide remains the leading cause of death among Australians aged 15 to 44 years.^1^ The economic burden of suicide is also high, with substantial financial and human costs in the form of medical care and forensic expenses, lost productivity, and psychosocial distress in family members and friends.^2^

Within Australia, 5% of all suicides in 2020 occurred by jumps from heights (eg, from bridges and cliffs).^1^ Despite the relatively low proportion of total suicides accounted for by this method, these sites are of high priority for several reasons. Suicide attempts involving jumping tend to be fatal, and suicides at these sites are often witnessed by bystanders.^3^ These sites also often gain reputations as places where people go to take their lives because of their accessibility and the media attention that often surrounds them.^3^

Means restriction via barriers has been shown to be an effective suicide prevention strategy at these sites.^3,4,5^ Despite this, there is sometimes considerable resistance to installing barriers, with one of the key arguments being cost. Relatively few studies have considered the cost-effectiveness of installing barriers for suicide prevention. Studies from the US^6^ and UK^7^ have indicated that the installation of barriers at bridges is highly cost-effective. However, these studies were limited to single sites and did not consider cliffs where suicides are also known to occur. No study has examined the cost-effectiveness of barriers on a national scale. To help guide policy decision-making on investments, we aimed to examine the cost-effectiveness of installing barriers at selected bridge and cliff sites across Australia over 5 and 10 years. The specific sites had previously been identified as being of concern in a national review that we conducted.^8^

## Methods

Our economic evaluation adhered to the Consolidated Health Economic Evaluation Reporting Standards (CHEERS) reporting guideline (eTable 1 in the Supplement). We conducted a secondary analysis of existing data,^3^ and therefore informed consent requirements were waived; ethics approval for the identification of Australian target sites was received from the University of Melbourne, Medicine and Dentistry human ethics subcommittee. We adopted a partial societal perspective (including intervention costs and monetary value associated with preventing suicide deaths) that aligns with the recommendation from the Second Panel on Cost-effectiveness in Health and Medicine.^9^ A simple decision tree model was developed to examine reductions in suicides if barriers were installed across identified bridge and cliff sites. We used both return on investment (ROI) and cost-utility analysis (CUA) frameworks to determine the economic credentials of barrier installation. The economic model required various inputs, including: (1) relative risk (RR) estimates of effectiveness of barriers (in terms of reductions in suicide compared with no barriers) at bridge and cliff sites, respectively; (2) RR estimates of substitution to nearby sites following barrier installation; (3) monetary value associated with preventing suicide deaths; and (4) costs of implementing and maintaining the intervention over 5 and 10 years. The analysis did not consider cost savings due to averting nonfatal suicide attempts because no data were available on these, which means that the analysis was conservative. The economic model was built in Excel version 2201 (Microsoft Corp) with the Ersatz add-in used to run Monte Carlo simulations.

### Setting and Target Population

In total, 7 bridges and 19 cliff sites were identified from a previous report examining locations where suicides are known to occur in Australia.^8^ These bridge and cliff sites were included on the basis that they were specific, accessible, and had been reported to have a high number of suicides previously (2 or more suicides over a 5-year period between 2012 and 2016).^8^ Across the 7 bridge sites, there were a total of 54 suicides over 5 years (approximately 10.8 suicides per year at these sites in 2016). Across the 19 cliff sites, there were a total of 83 suicides over 5 years (approximately 17 suicides per year in 2016). During the study period, most sites had no barriers installed. One bridge and 1 cliff site had fencing installed for suicide prevention prior to the study period. However, because there had been contention regarding the effectiveness of the barrier at the cliff site^10^ and both sites continued to report 2 or more suicides, they were therefore included in the study on the basis that the installed barriers were suboptimal. The target population were people who attempt suicide at these sites, regardless of age, race and ethnicity, or gender.

### Statistical Analysis

#### Association of Barriers With Suicide Rates

We updated a systematic review and meta-analysis on the effectiveness of barriers that was published in 2020 by Okolie et al.^5^ More specifically, we searched for studies that were published between January 2019 (the latest search date in the Okolie et al review) and November 2021 using search methods previously described in our own 2015 meta-analysis of barriers.^3^ Studies were included if the barrier was installed at a bridge (including terrace or viaduct) or cliff location and the main method of suicide at these locations was jumping. The intervention could be delivered in isolation or in combination with other measures (eg, crisis support signage, closed-circuit television surveillance). We identified some studies that reported on evaluations of barrier installation at the same site at different time periods. To avoid duplication in counts, studies that examined the same location were treated as a single study. All studies included in this analysis are presented in eTable 2 in the Supplement.

Pooled incidence rate ratios (IRR) were generated to estimate the changes in suicide rates associated with barrier installation at bridges (10 studies), cliffs (3 studies), and substitution to other nearby sites (10 studies). For bridges, as 1 study from Switzerland^11^ contained multiple effect sizes for different bridge sites, we allowed for an additional level of clustering (multiple estimates clustered within a single study) when calculating the pooled IRR. For cliff and substitution estimates, mixed-effects Poisson regression models were fit using methods described by Spittal, Pirkis, and Gurrin.^12^ We then applied the intervention effect size estimates within the Australian context to project the total reduction in suicides at the identified bridge and cliff sites, respectively. Meta-analyses were conducted in Stata version 16.1 (StataCorp) using the mepoisson command.

#### Monetary Savings

To estimate the monetary value society places on reducing the risk of death, the current study used the value of a statistical life recommended by the Australian Government Department of the Prime Minister and Cabinet.^13^ The value of a statistical life is estimated at US $3.1 million, and US $0.1 million for the value of a statistical life-year, both measured in 2018 US$. These estimates represented an average and were based on a healthy person living for another 40 years.^14^

#### Intervention Costs

Intervention costs for barrier installation were derived from Australian news sources.^10,15,16,17^ We calculated these costs by summing the initial installation costs and costs to maintain the barriers over 5 and 10 years. The mean initial costs to build a barrier at a bridge or cliff were estimated at US $10.5 million and US $1.6 million, respectively.^10,15,16,18^ Given the uncertainty of the unit cost to build a barrier, a 20% uncertainty was applied for the unit costs. We assumed that maintenance of a barrier could be broken down into cyclical and condition-based activities with minor maintenance after 5 years and major maintenance after 10 years.^17^ We assumed minor maintenance and major maintenance costs of 20% and 50% of total installation costs, respectively.

#### Cost-effectiveness Frameworks

We designated 2018 as the reference year, with a discount rate of 3% per year applied to all costs and health outcomes. All costs were expressed in US$ and converted to 2018 prices. All non-US$ currencies were converted to US$ currencies using the Cochrane Economics Methods Group–Evidence for Policy Practice Information and Coordinating Centre Cost Converter version 1.4 that uses the purchasing power parity approach sourced from the International Monetary Fund World Economic Outlook database.^19^

Our primary analysis adopted an ROI framework, which compared the cost savings produced by an intervention with the intervention costs. This ratio is technically a benefit-cost ratio. This differs from a conventional ROI ratio, which comprises net discounted cost savings (ie, total discounted cost savings minus total discounted costs) divided by total discounted costs. Our presentation of the benefit-cost ratio as a type of ROI ratio in this study is an approach used in previous ROI studies published by Australia’s National Mental Health Commission^20^ and Public Health England.^21^ Interventions with ROI ratios greater than 1 are deemed cost-effective.

Our secondary analysis was a cost-effectiveness analysis that was reported as an incremental cost-effectiveness ratio (ICER). The ICER comprised the difference in costs between the intervention and no barrier installed (the comparator) divided by the difference in reduction of suicide cases.

#### Uncertainty Analyses

We conducted uncertainty analyses alongside the ROI and cost-effectiveness models to propagate parameter uncertainty (ie, sampling error) from the input parameters to the final model outputs. We used a Monte Carlo simulation with 3000 iterations. Intervention costs, cost offsets, suicides, ROI ratios, and ICERs were estimated with accompanying 95% uncertainty intervals (95% UIs). For the cost-effectiveness analysis, uncertainty iterations were also represented on a cost-effectiveness plane that is commonly used in analyses of health sector interventions and includes 4 quadrants. In the northeast quadrant, the intervention is cost-effective if the ICER falls under the specified value-for-money criterion because the intervention is more effective and costlier than the comparator. In the southeast quadrant, the intervention is less costly and more effective than the comparator (ie, dominant); therefore, the intervention is likely to yield high value for money. In the southwest quadrant, the intervention is less costly and less effective; therefore, the decision to adopt the intervention may be based on decision-makers’ willingness to accept some health loss relative to cost saving. Finally, in the northwest quadrant, the intervention is associated with greater costs but less health gain, and therefore is not considered a viable option.

#### Sensitivity Analyses

We also conducted a series of sensitivity analyses (SAs) to test the robustness of the cost-effectiveness and ROI results to changes in the input parameters and/or assumptions. We conducted a threshold analysis where we reduced the number of suicides prevented by barrier installation until the intervention was not cost saving (ie, reflecting reduced effectiveness of intervention on suicides). We also assumed there was no annual increase in suicide at the sites every year instead of 3% increase in the base case. To capture the monetary value associated with suicide within the time horizon of the study, we used the value of a statistical life-year over 5 and 10 years rather than the (lifetime) value of a statistical life.

In the absence of precise estimates on maintenance costs, we factored in both low and high maintenance costs. For lower maintenance cost, we assumed that the cost reduces to 5% of total installation costs for minor maintenance cost and 20% for major maintenance cost. For higher maintenance cost, we assumed that the cost increases to 30% for minor maintenance cost and 60% for major maintenance cost.

## Results

### Association of Barriers With Rates of Suicide at Installation Sites and Sites Nearby

In the 10 studies conducted at bridges, the pooled estimates indicated that barrier installations were associated with an 84% reduction in the number of suicides per year (IRR, 0.16; 95% CI, 0.13-0.20) (Table 1). Using the intervention effect size, we estimated that in the Australian context, across the 7 bridge sites identified, the aggregate number of suicides would decrease from a mean of 11 to 2 suicides per year.

**Table 1.** Input Parameters and Uncertainty Ranges for Health Benefit and Costing Analysis of Barriers for Suicide Prevention

Parameter	IRR (95% CI)	Uncertainty distribution	Source(s)
Effect of barrier installation at bridge sites	0.16 (0.13-0.20)	Lognormal	Meta-analysis^11,22,23,24,25,26,27,28,29,30,31,32,33,34^
Effect of barrier installation at cliff sites	0.72 (0.50-1.05)	Lognormal	Meta-analysis^35,36,37,38^
Effect of barrier installation at nearby sites	1.01 (0.85-1.20)	Lognormal	Meta-analysis^22,25,26,27,28,29,30,31,32,33,34,36^
**Costing analysis**			
Unit cost, $			
Barriers at bridge	10 459 736	Fixed	Australian news^15,16^
Barriers at cliffs	1 638 115	Fixed	Australian news^10^
Uncertainty, %	±20	Pert	Technical reports^39,40^
Maintenance cost, % (range)			
Minor	20 (10-30)	Pert	Own assumption
Major	50 (40-60)	Pert	Own assumption
Statistical life year value, $	3 093 725	Fixed	Department of the Prime Minister and Cabinet^13^

For cliff sites, the data from the 3 available studies indicated there was no evidence of an association between barrier installation and suicides per year (IRR, 0.72; 95% CI, 0.50-1.05). Despite the lack of statistical evidence, 2 of the studies showed a reduction to zero following barrier installation,^35,36^ and the remaining study showed a 60% reduction.^37^ Given the consistent reduction in suicides, we used the pooled point estimate and assumed a 28% reduction in suicides from 18 to 13 suicides per year across the 19 cliff sites.

In the 10 studies where information on suicide at nearby sites was available for pooling, barrier installation was not associated with a change in the number of suicides per year at these neighboring sites (IRR, 1.01; 95% CI, 0.85-1.20) (Table 1). Given there was no evidence of an association at nearby sites and results were mixed across the 10 studies identified, we assumed no increase in suicides in the remaining identified sites where barriers were not installed.

### Cost-effectiveness of Barriers

The total cost was estimated at US $86 million (95% UI, $82 million to $91 million) and US $114 million (95% UI, $108 to $120 million) for installing barriers at 7 bridges over 5 and 10 years, respectively (Table 2). Installing barriers at bridges was associated with a reduction of 50 suicides (95% UI, 31 to 55) over 5 years and 100 suicides (95% UI, 65 to 110) over 10 years. The interventions produced corresponding monetary savings of US $145 million (95% UI, $90 to $160 million) over 5 years and US $270 million (95% UI, $176 to $298 million) over 10 years. Evaluating the intervention cost in relation to cost savings, the estimated ROI ratio was 1.7 (95% UI, 1.0 to 1.9) over 5 years and 2.4 (95% UI, 1.5 to 2.7) over 10 years (Table 2).

**Table 2.** Cost-effectiveness Summary for Installing Barriers at Bridge and Cliff Sites Where Suicides are Known to Occur in Australia

Output parameter	Cost, median (95% UI), $US in millions			
	Bridges		Cliff sites	
	5 y	10 y	5 y	10 y
Intervention costs	86 (82 to 91)	114 (108 to 120)	46 (41 to 50)	74 (67 to 80)
Cost offsets^a^	−145 (−90 to −160)	−270 (−176 to −298)	−78 (−147 to 44)	−145 (−274 to 82)
Net costs^a^	−58 (−2 to 75)	−156 (−58 to −184)	−32 (−102 to 89)	−71 (−201 to 154)
Suicides averted, No. (95% UI)	50 (31 to 55)	100 (65 to 110)	27 (−15 to 51)	53 (−30 to 101)
ROI ratio (95% UI)	1.7 (1.0 to 1.9)	2.4 (1.5 to 2.7)	1.7 (−0.9 to 3.3)	2.0 (−1.1 to 3.8)
ICER (95% UI)^b^	Dominant (dominant to dominant)	Dominant (dominant to dominant)	Dominant (dominant to dominated)	Dominant (dominant to dominated)

Abbreviations: ICER, incremental cost-effectiveness ratio; ROI, return on investment; UI, uncertainty interval.

^a^ Negative costs denote cost savings (if positive costs denote an expense).

^b^ A dominant ICER signifies that the intervention is both cost saving and produces greater positive health outcomes when compared with the comparator; a dominated ICER signifies that the intervention is more costly and produces less positive health outcomes when compared with the comparator.

The total cost for installing barriers at 19 cliff sites was US $46 million (95% UI, $41 to $50 million) and US $74 million (95% UI, $67 to $80 million) over 5 and 10 years, respectively. Installing barriers at cliff sites was associated with a reduction of 27 suicides (95% UI, −15 to 51) over 5 years, and 53 (95% UI, −30 to 101) suicides over 10 years. A monetary savings value was estimated at US $78 million (95% UI, $−44 million to $147 million) and US $145 million (95% UI, $−82 million to $274 million) over 5 and 10 years, respectively. There was no statistical evidence of cost savings in relation to intervention cost for cliff sites over 5 years (ROI, 1.7; 95% UI, −0.9 to 3.3) and 10 years (ROI, 2.0; 95% UI, −1.1 to 3.8) (Table 2). When evaluating the intervention cost in relation to reduction of suicides (the ICER), installing barriers at either bridges or cliffs was cost saving (ie, monetary savings due to averted suicides over the intervention cost) compared with no intervention (Table 2).

### Uncertainty Analysis

The cost-effectiveness plane indicated that for bridge sites, the likelihood of barriers being cost saving was 98% and 100% over 5 and 10 years, respectively (Figure). For cliff sites, the cost-effectiveness plane of installing barriers at cliff sites spreads over from the dominated quadrant (ie, more costly and less effective) to dominant quadrant (cost saving). Results indicated that the likelihood of barriers being cost saving at cliff sites was 73% and 75% over 5 and 10 years, respectively.

**Figure.**
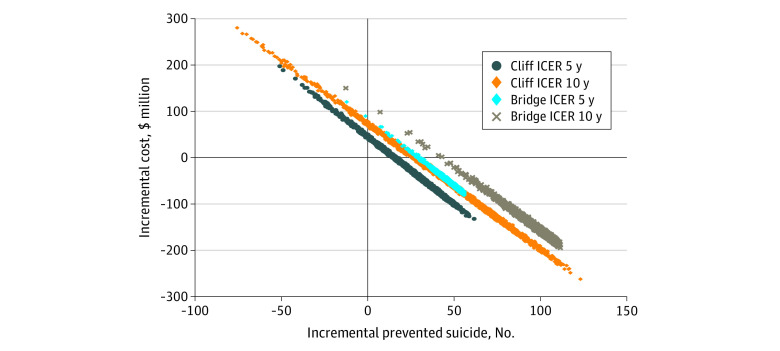
Cost-effectiveness Plane of Installing Barriers at Known Bridge and Cliff Sites Where Suicides Have Occurred in Australia

### Sensitivity Analyses

In the first SA, a threshold analysis indicated that building barriers at bridges would maintain cost saving status if there was a reduction of 30 and 41 suicides over 5 and 10 years (or approximately a reduction of 4 and 6 suicides per year), respectively. Regarding cliff sites, the intervention would be cost saving if at least 15 and 24 suicides were prevented at 19 cliff sites over 5 and 10 years (or approximately a reduction of at least 3 suicides per year), respectively. In the second SA, which assumed that suicides at bridges and cliffs remained unchanged every year, results were consistent with the baseline model (Table 3). In the third SA, where cost saving was estimated by the value of a statistical life year over 10 years, the ROIs reduced to 0.5 (95% UI, 0.3 to 0.6) for bridges and 0.4 (95% UI, −0.2 to 0.9) for cliffs (Table 3). Variations to maintenance costs of barriers showed that lowering maintenance costs (the fourth SA) increased the ROI of the intervention at bridges over 10 years to 3.1 (95% UI, 2.0 to 3.4). After increasing the maintenance costs (the fifth SA), ROI results were largely consistent with the baseline model (Table 3).

**Table 3.** ROI Results in Scenario Analysis

Scenario analyses	ROI ratio, $ (95% UI)^a^			
	5 y		10 y	
	Bridges	Cliff sites	Bridges	Cliff sites
Baseline model	1.7 (1.0 to 1.9)	1.7 (−0.9 to 3.3)	2.4 (1.5 to 2.7)	2.0 (−1.1 to 3.8)
No increased suicide every year	1.5 (1.0 to 1.7)	1.7 (−0.8 to 3.3)	2.0 (1.3 to 2.3)	2.0 (−1.0 to 3.8)
Cost saving estimated by the value of statistical life-year	0.2 (0.1 to 0.2)	0.2 (−0.1 to 0.4)	0.5 (0.3 to 0.6)	0.4 (−0.2 to 0.9)
Lower maintenance^b^	1.9 (1.2 to 2.1)	2.2 (−1.0 to 4.1)	3.1 (2.0 to 3.4)	3.2 (−1.5 to 5.9)
Higher maintenance^c^	1.6 (1.0 to 1.7)	1.5 (−0.8 to 2.9)	2.1 (1.4 to 2.4)	1.7 (−0.9 to 3.3)

Abbreviations: ROI, return on investment; UI, uncertainty interval.

^a^ ROI ratio indicates US$ saved per US $1 invested.

^b^ Estimated minor maintenance cost of 5% and major maintenance cost of 20% of total installation costs.

^c^ Estimated minor maintenance cost of 30% and major maintenance cost of 60% of total installation costs.

## Discussion

To our knowledge, this is the first national study examining the cost-effectiveness of installing barriers at multiple bridge and cliff sites where suicides are known to occur. In our analyses, the installation of barriers was a cost-effective means of preventing suicides at bridges in both the short-term (5 years) and the long-term (10 years). If 6 suicides were prevented each year over 5 years following barrier installation at bridges, the intervention would still be cost saving. In addition, cost-effectiveness was maintained even when the model assumed inflation in maintenance costs. Although similar ROI point estimates were found for cliff sites, the results were not statistically significant, possibly because of the limited studies available on barrier effectiveness at cliff sites. Further research is needed on barrier installation at cliff sites.

Installation of barriers constitutes a current best practice approach for reducing suicides in England, Scotland, and Australia.^41^ The findings of our national study expand on findings from previous, smaller economic evaluation studies of barriers constructed at key bridges in the UK^7^ and US.^6^ Knapp and colleagues^7^ examined the financial benefits of installing a barrier at a suspension bridge in the UK over 5-year and 10-year periods. Their analysis showed that installation of a barrier was cost saving over both time periods. Similarly, after installation of a safety barrier at the Golden Gate Bridge in San Francisco, a cost-benefit analysis indicated that the intervention was highly cost saving.^6^ Given country-level differences in model parameters used, effect size estimates across international studies vary; however, the conclusion remains the same—barriers are cost-effective.

### Strengths and Limitations

To our knowledge, this is the first multisite economic evaluation of barriers for suicide prevention. Our study makes important distinctions between cost-effectiveness at bridges and cliff sites, and accounts for variation in assumptions through uncertainty and sensitivity analyses. Our study also provides an update on the evidence of barrier effectiveness for suicide prevention at bridges, cliffs, and substitution effects at nearby sites.

Despite this, there are a number of important limitations that should be considered when interpreting these findings. The evidence for barrier effectiveness at cliff sites was limited to 3 studies, reducing statistical power for pooled analysis. Although all 3 cliff studies showed a reduction in suicides after barriers were installed, the lack of an association for barriers at cliffs in the pooled estimate and subsequent cost-effectiveness analysis for cliffs should be interpreted with caution. In addition, we were unable to factor cost savings associated with averted suicide attempts and unintentional injuries and fatalities (eg, accidental falls from cliff sites) into our analyses because of an absence of data, but this means that our estimate of the benefit of installing barriers is likely to be an underestimate.

Furthermore, our analysis assumed that the costs and benefits of installing barriers may be yet to accrue. Two sites (1 bridge and 1 cliff) had some form of barrier installed for suicide prevention prior to the 5-year study period, although the effectiveness of the barrier at the cliff site had been challenged because of its height.^10^ In addition, both sites continued to report 2 or more suicides per year and thus were identified as sites of concern in our previous study.^8^ Given the barriers at these sites were deemed suboptimal, it is likely they would have seen a reduction in suicides over 5 years of the kind of magnitude reported in our study if better barriers were installed. Consideration should be given to strengthening barriers at sites where barriers are identified as suboptimal to account for barrier height, coverage of jump area, and inward curvature to prevent climbing.^5,11^ Moreover, as the cost of barrier installation at identified bridge and cliff sites may vary (particularly if there are preexisting barriers), we applied a 20% uncertainty to the cost of barrier installation to account for this variation. The initial and ongoing cost of barriers are also presumably similar to what is reported in our study (in fact, the cost of installation was based on a barrier built at one of the identified sites), therefore this is unlikely to impact our study conclusions. Barrier installation is often accompanied by other relatively inexpensive preventive measures such as the installation of security cameras for surveillance, crisis support signage, and crisis telephone booths.^3^ Our results might also be partially affected by these measures. For this reason, the estimated benefit of the barrier alone would be biased upward.

Given there was no evidence of an increase in suicides at nearby sites from our pooled analysis, we did not consider substitution to other sites. Further work is needed to critically evaluate the substitution effect given the mixed evidence so that it can be factored into cost-effectiveness calculations. In addition, we were not able to account for substitution, where suicides occurred by different methods, because of an absence of data. Jumping from a height is a relatively rare method of suicide, so it would be difficult to demonstrate statistically that substitution to other methods (eg, hanging) had or had not occurred at a population level. Qualitative studies exploring what people do if their suicide attempt by one means is thwarted (ie, do they change their course of action for the better and seek help, or do they look for alternative means?) would be beneficial. Despite it being a relatively rare method of suicide and the potential risk of method substitution, preventing suicides at these sites is important because these deaths often have a longstanding effect on witnesses and others who may use the site. To mitigate concerns around substitution, we conducted a threshold analysis (ie, lowered the effectiveness of the intervention), which indicated the prevention of 6 suicides per year over 5 years would be enough for barriers at bridges to be cost saving. Notably, these estimates do not account for effects on family and friends of individuals who have died by suicide, therefore our estimates are conservative.

In addition, it is worth noting that the costs of installing barriers are likely to vary between countries, as are the numbers of suicides occurring at bridges and cliffs. Moreover, because of the absence of academic or government sources on costs of barrier installation, intervention unit costs were derived from Australian news sources. The simulation model used in this study, which was in line with all similar simulation models, should be interpreted in the light of the assumptions that underpin it. However, we note that we deliberately varied our assumptions in our uncertainty analysis and sensitivity analyses, and often our assumptions were quite conservative.

## Conclusions

Our study provides robust evidence of the cost-effectiveness of installing barriers at bridges, thus strengthening the evidence base for this suicide prevention strategy. Although we and others have previously demonstrated that barriers are effective, the implementation of this as a strategy has faced challenges, with cost often cited as a major stumbling block. The bulk of the intervention costs are associated with the initial installation. Although the initial cost may be high, our findings showed that even when subsequent maintenance costs were inflated, installing barriers at bridges remained a highly cost-effective intervention. This study did not find significant evidence for the effectiveness and cost-effectiveness of barrier installation at cliff sites, suggesting further research and a broader approach to suicide prevention (in addition to means restriction) at cliff sites is needed.

## Notes

Supplement. **eTable 1.** Completed Consolidated Health Economic Evaluation Reporting Standards (CHEERS) Checklist **eTable 2.** Pre- and Postbarrier Intervention Suicide Count for Studies Examining Bridge and Cliff Sites
