# Supplementary material for: Cost-effectiveness of Installing Barriers at Bridge and Cliff Sites for Suicide Prevention in Australia
Source: JAMA Netw Open. 2022 Apr 5;5(4):e226019. doi: 10.1001/jamanetworkopen.2022.6019 (PMC8984771; doi:10.1001/jamanetworkopen.2022.6019)
Supplement: Supplement. — eTable 1. Completed Consolidated Health Economic Evaluation Reporting Standards (CHEERS) Checklist eTable 2. Pre- and Postbarrier Intervention Suicide Count for Studies Examining Bridge and Cliff Sites [file jamanetwopen-e226019-s001.pdf]

Supplemental Online Content

Bandara P, Pirkis J, Clapperton A, et al. Cost-effectiveness of installing barriers at bridge and cliff sites for suicide prevention in Australia. *JAMA Netw Open.* 2022;5(4):e226019. doi:10.1001/jamanetworkopen.2022.6019

eTable 1. Completed Consolidated Health Economic Evaluation Reporting Standards (CHEERS) Checklist

eTable 2. Pre- and Postbarrier Intervention Suicide Count for Studies Examining Bridge and Cliff Sites

This supplemental material has been provided by the authors to give readers additional information about their work.

© 2022 Bandara P et al. *JAMA Network Open.*

eTable 1. Completed Consolidated Health Economic Evaluation Reporting Standards (CHEERS) Checklist

Section/item	Item No	Recommendation	Section reported
**Title and abstract**			
Title	1	Identify the study as an economic evaluation or use more specific terms such as "cost-effectiveness analysis", and describe the interventions compared.	See Title
Abstract	2	Provide a structured summary of objectives, perspective, setting, methods (including study design and inputs), results (including base case and uncertainty analyses), and conclusions.	See Abstract
**Introduction**			
Background and objectives	3	Provide an explicit statement of the broader context for the study.	See Introduction
		Present the study question and its relevance for health policy or practice decisions.	See Introduction
**Methods**			
Target population and subgroups	4	Describe characteristics of the base case population and subgroups analysed, including why they were chosen.	See the first paragraph in the Methods and Setting & target population
Setting and location	5	State relevant aspects of the system(s) in which the decision(s) need(s) to be made.	See *Setting* subsection in the Methods
Study perspective	6	Describe the perspective of the study and relate this to the costs being evaluated.	See the first paragraph in the Methods
Comparators	7	Describe the interventions or strategies being compared and state why they were chosen.	See the first paragraph in the Methods and Cost-effectiveness frameworks
Time horizon	8	State the time horizon(s) over which costs and consequences are being evaluated and say why appropriate.	See the first paragraph in the Methods
Discount rate	9	Report the choice of discount rate(s) used for costs and outcomes and say why appropriate.	See the first paragraph in the cost-effectiveness frameworks subsection the Methods
Choice of health outcomes	10	Describe what outcomes were used as the measure(s) of benefit in the evaluation and their relevance for the type of analysis performed.	See *Cost effectiveness model* subsections in Methods
Measurement of effectiveness	11a	*Single study-based estimates:* Describe fully the design features of the single effectiveness study and why the single study was a sufficient source of clinical effectiveness data.	Not applicable
	11b	*Synthesis-based estimates*: Describe fully the methods used for identification of included studies and synthesis of clinical effectiveness data.	See *Intervention effect size* subsection in the Methods
Measurement and valuation of preference based outcomes	12	If applicable, describe the population and methods used to elicit preferences for outcomes.	Not applicable
Estimating resources and costs	13a	*Single study-based economic evaluation:* Describe approaches used to estimate resource use associated with the alternative interventions. Describe primary or secondary research methods for valuing each resource item in terms of its unit cost. Describe any adjustments made to approximate to opportunity costs.	Not applicable
	13b	*Model-based economic evaluation:* Describe approaches and data sources used to estimate resource use associated with model health states. Describe primary or secondary research methods for valuing each resource item in terms of its unit cost. Describe any adjustments made to approximate to opportunity costs.	See *Intervention costs and monetary savings* subsection in the Methods

© 2022 Bandara P et al. *JAMA Network Open.*

Currency, price date, and conversion	14	Report the dates of the estimated resource quantities and unit costs. Describe methods for adjusting estimated unit costs to the year of reported costs if necessary. Describe methods for converting costs into a common currency base and the exchange rate.	See the *Cost-effectiveness frameworks* subsection in the Methods
Choice of model	15	Describe and give reasons for the specific type of decision-analytical model used. Providing a figure to show model structure is strongly recommended.	See the first paragraph in Methods
Assumptions	16	Describe all structural or other assumptions underpinning the decision-analytical model.	See Methods
Analytical methods	17	Describe all analytical methods supporting the evaluation. This could include methods for dealing with skewed, missing, or censored data; extrapolation methods; methods for pooling data; approaches to validate or make adjustments (such as half cycle corrections) to a model; and methods for handling population heterogeneity and uncertainty.	See Methods
**Results**			
Study parameters	18	Report the values, ranges, references, and, if used, probability distributions for all parameters. Report reasons or sources for distributions used to represent uncertainty where appropriate. Providing a table to show the input values is strongly recommended.	See Table 1 in the main manuscript
Incremental costs and outcomes	19	For each intervention, report mean values for the main categories of estimated costs and outcomes of interest, as well as mean differences between the comparator groups. If applicable, report incremental cost-effectiveness ratios.	See Table 2 in the main manuscript
Characterising uncertainty	20a	*Single study-based economic evaluation:* Describe the effects of sampling uncertainty for the estimated incremental cost and incremental effectiveness parameters, together with the impact of methodological assumptions (such as discount rate, study perspective).	Not applicable
	20b	*Model-based economic evaluation:* Describe the effects on the results of uncertainty for all input parameters, and uncertainty related to the structure of the model and assumptions.	See Results alongside Table 3 and Figure 1 in the main manuscript
Characterising heterogeneity	21	If applicable, report differences in costs, outcomes, or cost-effectiveness that can be explained by variations between subgroups of patients with different baseline characteristics or other observed variability in effects that are not reducible by more information.	Not applicable
**Discussion**			
Study findings, limitations, generalisability, and current knowledge	22	Summarise key study findings and describe how they support the conclusions reached. Discuss limitations and the generalisability of the findings and how the findings fit with current knowledge.	See Discussion
**Other**			
Source of funding	23	Describe how the study was funded and the role of the funder in the identification, design, conduct, and reporting of the analysis. Describe other non-monetary sources of support.	See the Funding declaration in Acknowledgements
Conflicts of interest	24	Describe any potential for conflict of interest of study contributors in accordance with journal policy. In the absence of a journal policy, we recommend authors comply with International Committee of Medical Journal Editors recommendations.	See the Declaration of conflicting interests in Acknowledgements

eTable 2. Pre- and Postbarrier Intervention Suicide Count for Studies Examining Bridge and Cliff Sites

	Observation period (years)		Total suicides at site		Total suicides at other sites	
**Lead author and year**	**Pre-intervention**	**Post-intervention**	**Pre-intervention**	**Post-intervention**	**Pre-intervention**	**Post-intervention**
**Bridges**						
Beautrais (2001), Beautrais (2009)§	6	4	19	0	12	7
Bennewith (2007), Bennewith (2011)	5	5	41	20	31	42
Law (2014)	4	19	20	22	21	87
Lester (1993), O'Carroll (1994)	7	5	25	1	12	10
Pelletier (2007)	22	22	14	0	9	9
Perron (2013)	13.5	5	135	13	107	30
Reisch (2005)	3	3	7	0	12	13
Sinyor (2017), Sinyor (2010)	11	11	105	1	111	121
Hemmer (2017) – site A	17.9	6.1	54	6		
Hemmer (2017) – site D	13.9	10.1	45	10		
Hemmer (2017) – site E	2	3.5	24	2		
Hemmer (2017) – site F	22	2	16	0		
Hemmer (2017) – site K	10.9	2.1	8	0		
Hemmer (2017) – site M	13	11	5	3		
Hemmer (2017) – site H	15	5	13	0		
Hemmer (2017) – site B	16	4	53	1		
Hemmer (2017) – site C	16	4	37	3		
Hemmer (2017) – site O	13.2	10.8	6	3		
Hemmer (2017) – site N	14.4	9.6	7	3		
Hemmer (2017) – site I	20.8	3.3	25	3		
Hemmer (2017) – site J	4	15	9	0		
Hemmer (2017) – site G	16	2.5	14	1		
Berman (2021)	6	30	17	4	21	59
**Cliffs**						
Isaac (2005)	14	0.4	221	0		
Lockley (2014), Ross (2020)	12	5	86	34		
Skegg (2009)	10	2	13	0	4	0

§Note: missing data from other sites between 1992-1994, 1998-2000, so pre and post-intervention observation period for other sites is 2 years, respectively.

© 2022 Bandara P et al. *JAMA Network Open.*
